# Establishment and validation of a predictive model for severe pneumonia in children

**DOI:** 10.4314/ahs.v25i1.14

**Published:** 2025-03

**Authors:** Wenhua Ye, Jinyan Wu, Mi Cao, Zaidong Yang

**Affiliations:** Department of Pediatric Intensive Care Medicine, Foshan Women and Children Hospital, Foshan, China

**Keywords:** Predictive model, severe pneumonia, children

## Abstract

**Background:**

This study aimed to develop a model for the early identification of severe pneumonia in children by comparing common laboratory indicators between children with ordinary pneumonia and severe pneumonia.

**Methodology:**

Children aged 1 month to 14 years, diagnosed with pneumonia and admitted to our hospital between January 2017 and June 2022, were included in the study. Participants were divided into two groups based on the severity of their pneumonia. Data, including demographic information, medical history, clinical symptoms, laboratory indicators, and treatment outcomes, were collected from the hospital's medical records system.

**Results:**

The single-factor analysis revealed significant differences (P < 0.05) between the two groups in various parameters, including age, length of hospital stay, repeated hospitalization within 90 days, invasive ventilation, Intensive Care Unit (ICU) stay time, birth history, temperature, respiratory rate, blood pressure, procalcitonin, and C-reactive protein. Binary logistic regression analysis indicated that high body temperature and high respiratory rate were independent risk factors for severe pneumonia (P < 0.05).

**Conclusion:**

A predictive model for severe pneumonia in children can identify the risk of progression to severe disease, enabling prompt treatment and improving patient prognosis. This reduces the burden on families and social security.

## Introduction

Pneumonia is a common respiratory tract infection among children, especially infants and young children. It can be classified into mild and severe categories based on disease severity^1^,^2^. Mild pneumonia is characterized by mild symptoms, the absence of complications, and a favorable prognosis. Conversely severe pneumonia presents with a rapid onset, significant changes, severe symptoms, and multiple complications, often posing a life-threatening risk to children^3^. In fact, it is a leading cause of death in children under the age of 5. Therefore, early identification and prediction of severe pneumonia in children are crucial for saving lives^4^.

The aim of this study is to develop a model capable of effectively identifying severe pneumonia in children at an early stage. This will be achieved by comparing common laboratory indicators between children with ordinary pneumonia and those with severe pneumonia. Currently, the severity grading of pediatric pneumonia primarily relies on modified consensus guidelines for managing community-acquired pneumonia in adults established by the Infectious Diseases Society of America or the American Thoracic Society. This approach is supplemented with early warning scores for children^5^. However, this method incorporates subjective elements and is not convenient for practical use in a clinical setting. Additionally, it is primarily based on adult standards and heavily relies on the physician's judgment, lacking objectivity. Consequently, there is a shortage of predictive models for severe pneumonia in children.

Therefore, the main objective of this study is to establish a model that can promptly identify severe pneumonia in children using commonly assessed laboratory indicators. By comparing the differences between children with ordinary pneumonia and those with severe pneumonia, this model aims to facilitate rapid clinical decision-making for healthcare professionals. This approach will enhance the objectivity and efficiency of diagnosing severe pneumonia in children, ultimately contributing to better patient outcomes.

## Methods

### Study Population

The study included children between the ages of 1 month and 14 years who were admitted to Foshan Maternal and Child Health Hospital for pneumonia between January 2017 and June 2022. To ensure standardized classification, community-acquired pneumonia in children was divided into two groups: severe pneumonia and ordinary pneumonia. The classification criteria were based on relevant literature's diagnostic criteria for severe pneumonia. Children with blood system diseases, malignant tumors, severe liver and kidney diseases, autoimmune diseases, and other conditions that could potentially affect the severity of pneumonia (such as congenital heart disease or severe trauma) were excluded from the study to maintain sample homogeneity. The research plan received approval from the Ethics Committee of Foshan Maternal and Child Health Hospital, adhering to the ethical standards outlined in the Helsinki Declaration of medical ethics.

### Data Collection

The study subjects were divided into a severe pneumonia group and an ordinary pneumonia group based on the diagnosis of severe pneumonia. Patient information such as name, gender, age in months, length of hospital stay, occurrence of repeated hospitalizations within 90 days, mortality, use of invasive ventilation, ICU stay, birth history, temperature, respiration rate, and admission blood pressure were collected from the hospital's medical record information system. Laboratory indicators on the day of admission for both groups were recorded by referring to the clinical laboratory records. The laboratory indicators included procalcitonin (PCT), C-reactive protein (CRP), activated partial thromboplastin time (APTT), and uric acid. Blood routine indicators were measured using the TEK8520 automatic 5-category blood cell analyzer (Jinan, China), and serum indicators were measured using the Olympus AU400 automatic biochemical analyzer (Tokyo, Japan).

### Statistical Analysis

Data was entered into Excel 2010 using double-entry, and Statistical Product and Service Solutions (SPSS) 25.0 software was used for statistical analysis. One-way ANOVA and multiple factor analysis were performed using SPSS 25.0 software. Normally distributed metric data was presented as mean ± standard deviation (mean ± SD), and independent sample t-tests were used for group comparisons. Non-normally distributed metric data was described using median and quartile range, and the non-parametric Mann-Whitney U test was used for group comparisons. Pearson's chisquare test was used for comparison of count data. Receiver operating characteristic (ROC) curves were used to evaluate the operational characteristics of the subjects. Statistical significance was set at P < 0.05 to indicate significant differences.

## Results

### Univariate analysis of severe pneumonia

The univariate analysis revealed statistically significant differences (P < 0.05) between the two groups in various factors. These factors included age, length of stay, rehospitalization within 90 days, invasive ventilation, Intensive Care Unit (ICU) stay, birth history, body temperature, respiration, blood pressure, procalcitonin (PCT), and C-reactive protein (CRP). However, there were no significant differences in gender, mortality, activated partial thromboplastin time (APTT), and uric acid levels between the two groups (P > 0.05) (Table 1).

**Table 1:** Univariate analysis of each variable in the severe pneumonia group and the common pneumonia group

Entry items		Severe pneumonia (n/%)	Non-severe pneumonia (n/%)	X2/Z/t	P
gender	man	291(64.81)	91 (61.48)	0.534	0.465
	woman	158(35.19)	57(38.52)		
age		10.00(3.00.24.00)	14.50(7.00,36.00)	-4.193	<0.001
Length of stay	>7	335(25.39)	20(13.51)	172.375	<0.001
	≤7	114(74.61)	128(86.49)		
90 days of repeated	Yes	0(0.00)	28(18.92)	89.126	<0.001
hospitalization	No	449(100.00)	120(81.08)		
Death	Yes	5(1.11)	0(0.00)	0.592	0.442
	No	444(98.89)	148(100.00)		
Invasive ventilation	Yes	280(62.36)	0(0.00)	173.82	<0.001
	No	169(37.64)	148(100.0)		
Stay in ICU	Yes	167(37.19)	0(0.00)	173.82	<0.001
	No	282(62.81)	148(100.00)		
History of pregnancy and dilivery	Unspecificness	321(72.30)	130(87.84)	17.486	0.002
	Premature, very low/low weight baby	76(17.12)	9(6.08)		
	Intrauterine distress and asphyxia	6(1.35)	2(1.35)		
	There is a preexisting disease	5(4.05)	0(0.00)		
	Premature delivery with intrauterine distress	23(5.18)	7(4.73)		
Body temperature		36.90(36.60,37.80)	36.80(36.50,37.20)	-2.635	0.008
Breathing		46.00(35.00,55.00)	28.00(25.00,32.00)	-13.685	<0.001
mmHg	Low	41(9.19)	6(4.06)	6.533	0.038
	Normal	388(87.00)	140(94.59)		
	High	17(3.81)	2(1.35)		
PCT		0.23(0.10,1.22)	0.21(0.10,0.58)	-1.401	0.161
CRP		9.40(2.70,25.70)	4.30(1.75,16.23)	-3.496	<0.001
APTT		42.35(38.10,46.38)	42.45(37.78,46.03)	-0.275	0.783
Uric Acid		239.00(193.25,310.50)	238.00(174.50,309.50)	-0.940	0.347

### Binary logistic regression analysis of severe pneumonia in children

Based on the significant factors identified in the univariate analysis (P < 0.05), a collinearity analysis was conducted to avoid multicollinearity issues. Hospital stay, readmission within 90 days, and ICU admission showed variance inflation factors exceeding 5 and were consequently excluded from further analysis. The remaining predictive factors were included in the binary logistic regression analysis, with the occurrence of severe pneumonia as the dependent variable (Table 2). The results of the logistic regression analysis showed that high body temperature (OR = 1.100, 95% CI: 1.033-1.170) and high respiratory rate (OR = 4.071, 95% CI: 1.355-12.231) were identified as independent risk factors for severe pneumonia in children (P < 0.05). However, age, systolic blood pressure at admission, C-re-active protein (CRP), and procalcitonin (PCT) did not show significant differences (P > 0.05), as shown in Table 3.

**Table 2:** Assignment of argument variables

Variable	Assignment specification
Severe pneumonia	Non-severe pneumonia =0, severe pneumonia =1
Age	Original value
History of pregnancy and dilivery	Yes =1, no =0
Body temperature	Original value
Breathing	Original value
Systolic blood pressure on admission	1= “Low”, 2= “Normal”, 3= “High
CRP	Original value
PCT	Original value

**Table 3:** Binary logistic regression analysis of children with severe pneumonia

Variable	B	S.E.	Wald	*P*	OR	95% confidence interval
Age	0.009	0.008	1.123	0.289	1.009	0.993-1.025
History of pregnancy and dilivery	1.243	0.512	5.890	0.015	3.467	1.270-9.463
Body temperature	0.677	0.193	12.254	<0.001	1.968	1.347-2.874
Breathing	0.192	0.027	49.035	<0.001	1.212	1.148-1.279
Systolic blood pressure on admission	0.17	0.027	0.086	0.769	1.189	0.374-3.772
CRP	0.011	0.007	2.536	0.111	1.011	0.997-1.025
Constant (quantity)	-31.893	7.529	17.945	<0.001	<0.001	

### ROC curve analysis of body temperature, respiration, and CRP for identifying severe pneumonia in children

**Figure 1:**
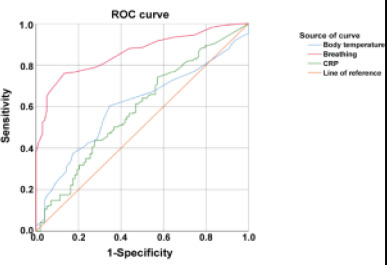
ROC curve of severe pneumonia in children

Receiver operating characteristic (ROC) curve analysis was performed to evaluate the predictive ability of the binary logistic regression model for severe pneumonia in children, using body temperature, respiration rate, and C-reactive protein (CRP) as independent variables. The results showed that the area under the curve (AUC) for body temperature was 60.39%, indicating moderate predictive performance. The AUC for respiration rate was 85.94%, indicating high predictive performance. Lastly, the AUC for CRP was 58.85%, indicating fair predictive performance (see Figure 1).

## Discussion

Childhood pneumonia is a common and frequently occurring disease in children. It progresses rapidly and is the leading cause of death among children^6^. Severe pneumonia accounts for 7% to 13% of all pneumonia cases in children. In China, as a densely populated developing country, severe pneumonia in childhood imposes a significant economic and social burden on families and society. Research has shown that severe pneumonia in children under 5 years old contributes to 0.86% of all child deaths annually in China, making the prevention and treatment of this disease a critical challenge^7^.

Currently, the most commonly used tools for predicting severe pneumonia in children in clinical practice include altered mental status, blood urea nitrogen, respiratory rate, blood pressure, and the pneumonia severity index. However, these evaluation criteria are mainly applied to adults, and the subjective judgment combined with laboratory tests used may not be suitable for diagnosing severe pneumonia in pediatrics. Therefore, early identification and assessment of the risk of childhood pneumonia progressing to severe disease, along with timely monitoring and effective treatment, can improve the prognosis of affected children and reduce the pressure on social security and family burden^8^.

The single-factor analysis revealed statistically significant differences (P < 0.05) between the two groups in various factors, including age, length of hospital stay, rehospitalization within 90 days, invasive mechanical ventilation, ICU admission, birth history, body temperature, respiratory rate, blood pressure, procalcitonin (PCT), and C-reactive protein (CRP).

As children grow older, their immune system gradually strengthens, reducing the probability of respiratory diseases and the development of severe pneumonia. This finding is consistent with previous research. A longer hospital stay or repeated hospitalizations indicate an unstable condition requiring further treatment and care. Invasive mechanical ventilation is used to improve oxygenation and ventilation, correcting hypoxemia and hypercapnia. Children requiring invasive mechanical ventilation usually exhibit more severe hypoxia, indicating a higher likelihood of developing severe pneumonia.

Moreover, children with a birth history have a higher probability of developing severe pneumonia compared to those without such a history. This finding aligns with multiple studies, as children born prematurely, with low birth weight, immunodeficiency, or congenital heart disease, have relatively weaker immune systems. In the case of lung infections, their ventilation function may be further compromised, promoting the development of pneumonia and ultimately leading to severe pneumonia.

Body temperature, respiratory rate, and blood pressure are vital signs that indicate the severity and criticality of a patient's condition. Therefore, when a patient develops severe pneumonia, it signifies that the condition has progressed to a more severe and critical stage, consistent with previous findings. The concentrations of PCT and CRP are positively correlated with the severity of the inflammatory response and can serve as markers for the early diagnosis of sepsis. This observation aligns with multiple studies^8^.

Overall, these findings highlight the significance of various factors in the development and severity of pneumonia in children, providing valuable insights for clinical practice and further research.

There are limitations to this study. First, the cases included in this study were from a single hospital in Foshan City, and data from other hospitals were not included in the analysis, making external validation impossible. Therefore, further expansion of the sample size is needed to improve the accuracy of the prediction model. Second, other factors that may affect the disease, such as body mass index, white blood cell count, acute physiology and chronic health evaluation II (APACHE II), and lactate dehydrogenase, were not included in the analysis. Third, some predictive factors (such as whether the hospital stay was longer than seven days and whether the patient had been rehospitalized within 90 days) overlap with each other. Therefore, further multicenter, large-sample, prospective studies are needed in different hospitals to enhance the accuracy and adaptability of the model.
